# Monitoring of seven industrial anaerobic digesters supplied with biochar

**DOI:** 10.1186/s13068-021-02034-5

**Published:** 2021-09-19

**Authors:** Kerstin Heitkamp, Adriel Latorre-Pérez, Sven Nefigmann, Helena Gimeno-Valero, Cristina Vilanova, Efri Jahmad, Christian Abendroth

**Affiliations:** 1 BioEnergie Verbund e.V, Jena, Germany; 2 Darwin Bioprospecting Excellence, S.L. Parc Cientific Universitat de Valencia, Paterna, Valencia Spain; 3 LUCRAT GmbH, Steinfurt, Germany; 4 Institute of Waste Management and Circular Economy, Technische Universität Dresden, Pirna, Germany; 5 Robert Boyle Institut e.V, Jena, Germany

**Keywords:** Anaerobic digestion, Biochar, DIET, Microbial communities

## Abstract

**Background:**

Recent research articles indicate that direct interspecies electron transfer (DIET) is an alternative metabolic route for methanogenic archaea that improves microbial methane productivity. It has been shown that multiple conductive materials such as biochar can be supplemented to anaerobic digesters to increase the rate of DIET. However, the industrial applicability, as well as the impact of such supplements on taxonomic profiles, has not been sufficiently assessed to date.

**Results:**

Seven industrial biogas plants were upgraded with a shock charge of 1.8 kg biochar per ton of reactor content and then 1.8 kg per ton were added to the substrate for one year. A joint analysis for all seven systems showed a decreasing trend for the concentration of acetic acid (*p* < 0.0001), propionic acid (*p* < 0.0001) and butyric acid (*p* = 0.0022), which was significant in all cases. Quantification of the cofactor F420 using fluorescence microscopy showed a reduction in methanogenic archaea by up to a power of ten. Methanogenic archaea could grow within the biochar, even if the number of cells was 4 times less than in the surrounding sludge. 16S-rRNA gene amplicon sequencing showed a higher microbial diversity in the biochar particles than in the sludge, as well as an accumulation of secondary fermenters and halotolerant bacteria. Taxonomic profiles indicate microbial electroactivity, and show the frequent occurrence of *Methanoculleus*, which has not been described in this context before.

**Conclusions:**

Our results shed light on the interplay between biochar particles and microbial communities in anaerobic digesters. Both the microbial diversity and the absolute frequency of the microorganisms involved were significantly changed between sludge samples and biochar particles. This is particularly important against the background of microbial process monitoring. In addition, it could be shown that biochar is suitable for reducing the content of inhibitory, volatile acids on an industrial scale.

**Supplementary Information:**

The online version contains supplementary material available at 10.1186/s13068-021-02034-5.

## Background

Anaerobic digestion is a methane-yielding process carried out by a microbial biocenosis composed of bacteria and methanogenic archaea. Firstly, substrate is hydrolyzed by bacteria. Further degradation by acetogenic bacteria leads to the formation of mainly organic acids, alcohols, hydrogen, and carbon dioxide. Eventually, the aforementioned metabolites are transformed into acetate, hydrogen, and carbon dioxide during acetogenesis. Metabolites produced by acetogenic bacteria are transformed by methanogenic archaea into methane [1]. Methanogenesis is usually divided into three major pathways: acetoclastic, hydrogenotrophic and methylotrophic methanogenesis [2]. In all three pathways, acetate, format, hydrogen and several methyl compounds (mono-, di- and trimethylamines) serve as electron carriers for a unique kind of respiration that uses carbon dioxide as electron acceptor [3]. If electrons are transported with the aforementioned carriers, this process is also referred to as mediated interspecies electron transfer (MIET). However and as discussed in a recent review article, more recent articles show that electrons can also be transported by conductive particles, direct cell contact or microbial nanowires [4]. This more direct way of electron transport is known as direct interspecies electron transfer (DIET) [4]. To increase the electroactivity of anaerobic digester microbiomes, multiple researchers have presented the possibility to increase the rate of DIET by adding conductive particles. In the past years, there has been a gold rush in the search for suited supplements. A particularly exotic one has recently been presented based on phenazine crystals [5]. It has been shown that phenazine crystals can form long and needle-like conductive structures, which overgrew with methanogenic archaea during the respective experiments.

A recent review by Martins et al. gives a detailed overview on many substances that have been applied to increase electroactivity, and the most popular ones are magnetite, hematite, granular activated carbon, carbon cloth and biochar [6]. The exact mechanisms of DIET and its impact on anaerobic digester communities are still under investigation. However, the first mechanisms have already been proposed. According to an article by Zhang et al., DIET contributed to lower hydrogen partial pressures, which in turn lowered the concentration of butyric acid [7].

“*Syntrophism among Prokaryotes*” is described extensively in a review article by Schink and Stams (2006). There are a variety of substrates that are syntrophically degraded, such as ethanol, butyrate, propionate, acetate and some amino acids and aromatics. Many syntrophic reactions release hydrogen [8]. Enzymatic reactions are usually bidirectional [9], and the direction that releases hydrogen is thermodynamically unfavoured for the abovementioned substrates. However, the hydrogen-releasing reaction occurs in spite of slightly endergonic reactions. Due to its poor solubility, hydrogen degasses rapidly from aqueous solutions, which prevents a hydrogen-consuming backreaction. The syntrophic partner organisms of such reactions contribute to low hydrogen pressures as they consume hydrogen in an exergonic reaction and very fast. Summing up both syntrophic reactions—the hydrogen producing and the hydrogen consuming—the resulting reaction is exergonic.

As the hydrogen-releasing reaction is thermodynamically unfavoured, it is very sensitive to hydrogen pressures. If hydrogen consumption is inhibited, or if hydrogen production is too fast, a slight increase in the hydrogen pressure might take place and inhibit the syntrophic degradation [8]. As conductive particles allow direct electron transport without the need for hydrogen interspecies transfer (HIT), this explains the enhancement in syntrophic butyric acid degradation described by Zhang et al., as previously mentioned.

Although the basic concept of syntrophy is well understood in anaerobic digestion, there is still much to learn about it. To give here an example of the underlying complexity, a recent study presented a model in which Clostridia, *Syntrophomonas*, *Methanosaeta* and hydrogenotrophic methanogens are intertwined [10]. Hydrolytic Clostridia produce fatty acids and acetate, and these fatty acids are further transformed to acetate by the acetogenic bacterium *Syntrophomonas*. Acetate is converted into carbon dioxide and methane by the acetoclastic methanogen *Methanosaeta* (*Methanothrix*) and, together with hydrogen, the produced carbon dioxide can then be converted to methane by hydrogenotrophic methanogens. However, it is also possible for *Methanothrix* to reduce carbon dioxide itself, using a direct inflow of electrons. Using ferroferric oxide, this inflow of electrons might be generated by *Syntrophomonas* during the acetogenic degradation of fatty acids. During the breakdown process, electrons are released, which are transferred to *Methanosaeta* by ferroferric oxide-assisted DIET [10]. The aforementioned microbial community indicates that direct and indirect transfers of electrons are microbiologically intertwined.

Additionally, it is poorly understood how conductive particles affect taxonomic profiles in anaerobic digesters. In the past few years, several articles have been published which highlight electroactive prokaryotes that are meaningful for anaerobic digesters. Important electroactive bacteria are, for example, the genera *Shewanella* [11] and *Geobacter* [12]. Among archaea, the acetoclastic methanogens *Methanosarcina* and *Methanothrix* appear to be important [6], and a recent study demonstrated that methanogenic archaea can form electrically conductive protein filaments, in particular, the hydrogenotroph methanogen *Methanospirillum hungatei* [13]. Also, it has been recently reported that the *Methanobacterium* strain YSL is able to form syntrophic aggregates with *Geobacter metallireducens *[14]. Altogether, recent information about DIET indicates that electroactivity occurs in a wide range of organisms within anaerobic digester microbiomes. As DIET seems to be a common phenomenon, which additionally allows enhancement of anaerobic digester microbiomes, this topic is of high interest for the biogas industry. However, to our best knowledge, there are no or very scarce studies that investigate the effect of conductive particles on industrial anaerobic digester microbiomes. The present study aims to close this gap. Therefore, seven different industrial digesters were analyzed with F420 fluorescent microscopy upon addition of large amounts of biochar. One digester was investigated in detail based on 16S-rRNA gene amplicon high-throughput sequencing. It has to be highlighted that not only DNA from sludge samples was analyzed, but also from biochar particles that were collected from fresh digestate.

## Results and discussion

### Influence of biochar on the spectrum of organic acids

**Fig. 1:**
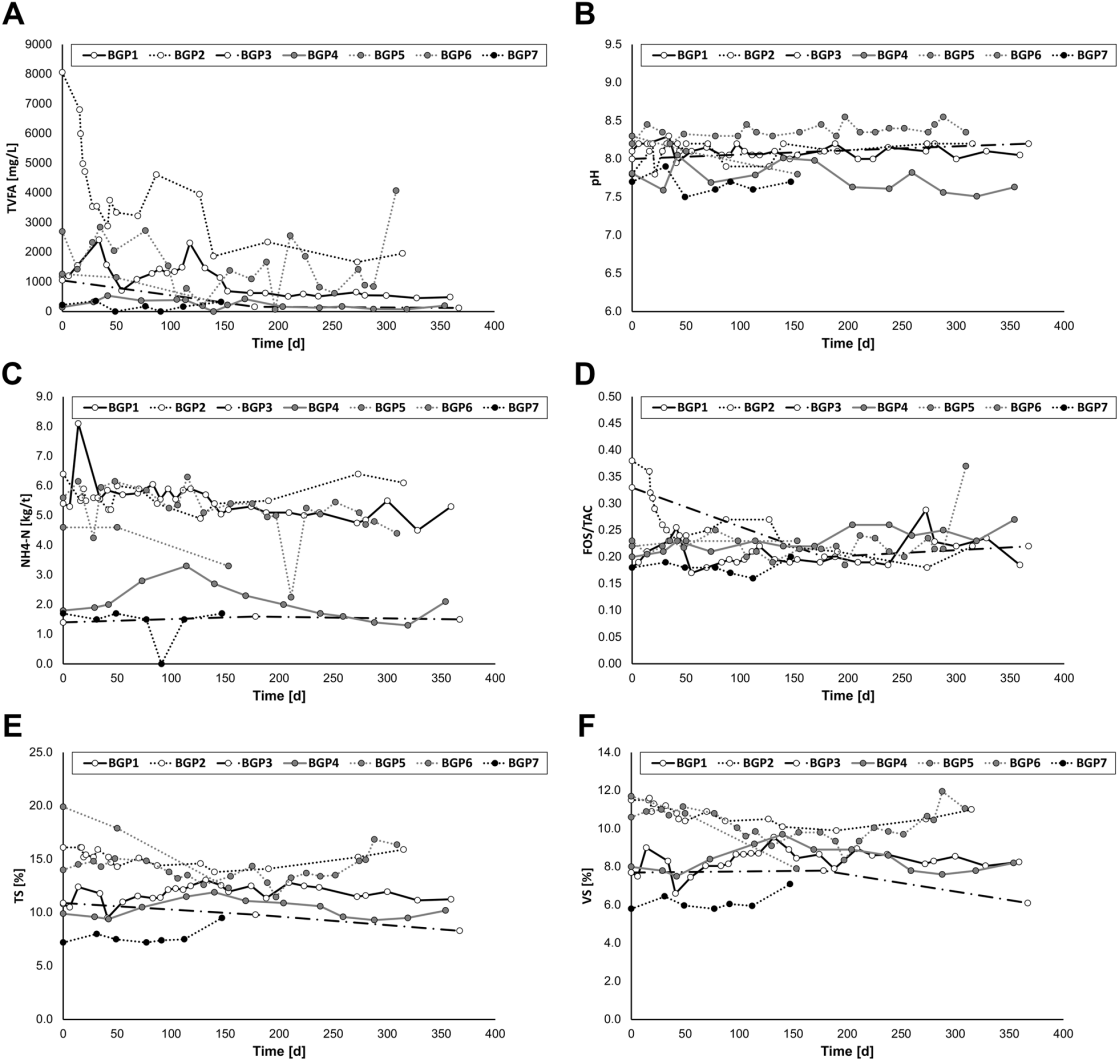
Chemical parameters collected by the plant operators: total volatile fatty acids (TFVAs, **A**); pH (**B**); ammonium nitrogen (NH4-N, **C**), FOS/TAC (**D**); total solids (TS) as percent of fresh biomass (**E**) and volatile solids (VS) as percent of TS

At the beginning of the project, seven customers (BGP1–BGP7) were acquired who, when using biochar in the context of the present study, agreed to allow insight into process data during the upgrade of the anaerobic digester plants (as described in Material and methods). At first, only BGP2 and BGP6 were in a critical condition reaching very high concentrations of total volatile fatty acids (TVFAs; 8059 mg L^−1^ of TVFAs for BGP2 and 2696 mg L^−1^ for BGP6). BGP1, BGP2-BGP5 and BG7 had TVFA concentrations lower than 2000 mg L^−1^. Some authors provide general information about inhibition thresholds from TVFAs. To give here an example: It was recommended that when using leftover food as a substrate, the amount of TVFAs should remain below 4000 mg L^−1^ [15]. However, the inhibition threshold varies depending on the situation at hand. The point at which an organic acid has an inhibiting effect depends considerably on the buffer capacity, the chemical composition, pH value and the type of acid [15]. According to Kroiss, a 10% inhibition is reached at acetic acid concentrations of approximately 1000 mg L^−1^. If the concentration is increased further to 3000 mg L^−1^, there is already a 50% inhibition. [16] Unlike acetic acid, propionic acid has a very strong inhibiting effect. At a pH of 6.5, a concentration of 150 mg L^−1^ propionic acid is already alarming. Under the same conditions, an equally strong inhibitory effect for acetic acid would only be expected at 1000 mg L^−1^ [17]. In this context, it is particularly alarming that some of the analyzed systems (BGP2-5) showed high levels of propionic acid at the beginning of the experiment (Additional file 1). Propionic acid concentrations were particularly high for BGP2 (1800 mg L^−1^) and BGP6 (1915 mg L^−1^). The fact that biogas was still formed at such high concentrations of propionic acid can be explained by the high pH value (between 7.5 and 8.0 for all plants; Fig. 1B). With a pK_S_ value of 4.87, propionic acid is almost completely deprotonated at pH values > 7.5. Protonated acids in particular are inhibitory, and the degree of protonation increases with a low pH value. The fact that pH values between 7.5 and 8.0 were maintained despite such high acid concentrations could be explained by an increased buffer capacity due to high NH_4_-N contents (Fig. 1C). With NH_4_-N concentrations ≥ 5 kg t^−1^, BGP1, BGP6 and BGP2 in particular showed high values.

Although no reliable dataset for the biogas productivity was given, all operators have regularly commissioned suitable service providers for chemical analyses, as described in Material and methods and Fig. 1. All raw data are provided in the Additional file 1. Most of the raw data yielded no meaningful interpretation. Although small variations were observed for pH, NH_4_-N, FOS/TAC, total solids (TS) and volatile solids (VS), no clear trend was observed for the respective parameters (Fig. 1B–F).

**Fig. 2:**
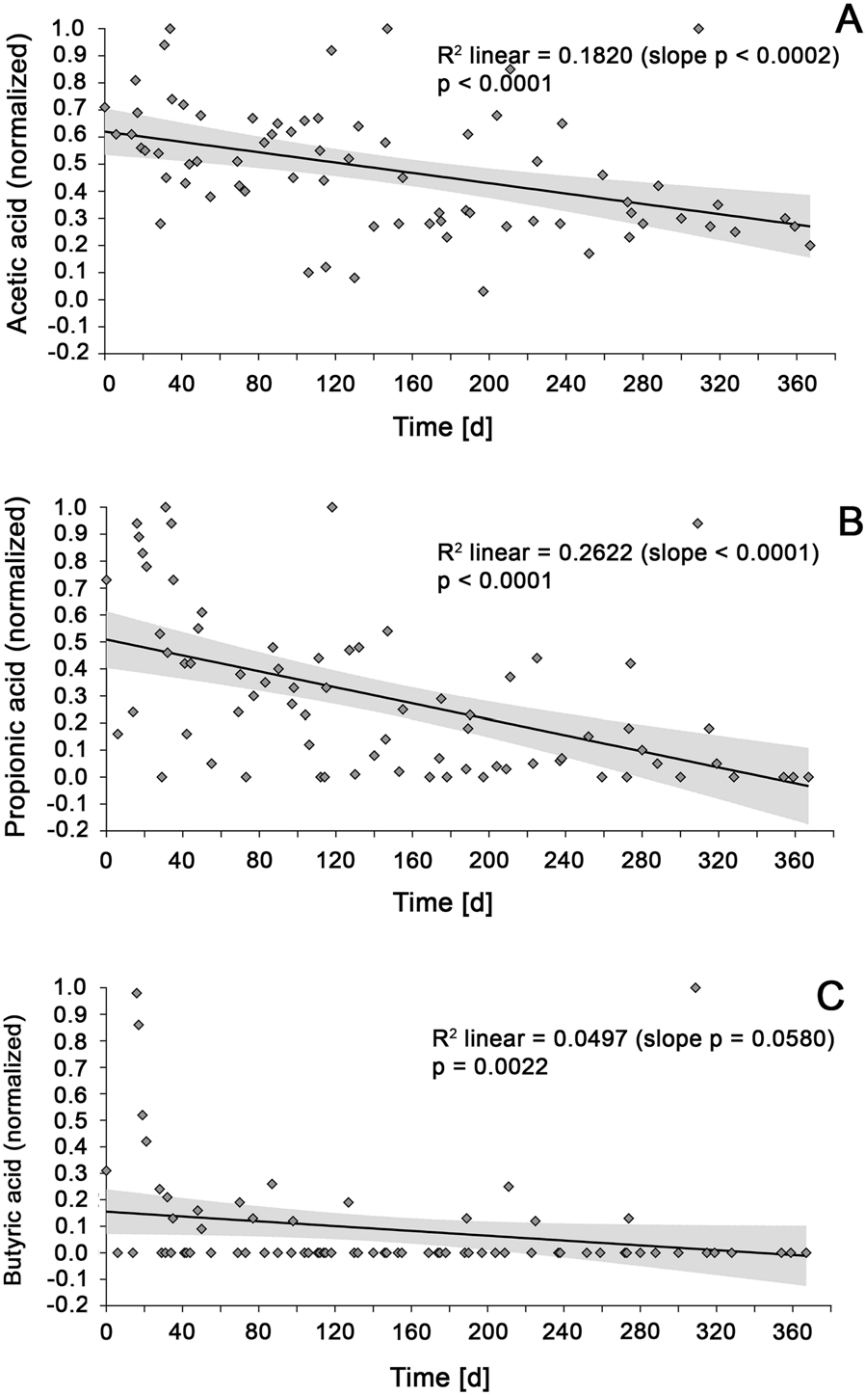
Evolution of organic acid concentration upon biochar supplementation: after normalization to a value between zero and one, mean values were calculated for all seven digesters (BGP1–BGP7). Concentrations were recorded over a duration of one year. The significance of the decrease in organics acids was assessed for acetic acid (**A**), propionic acid (**B**) and butyric acid (**C**) applying a nonparametrical Spearman test

However, upon biochar supplementation a decrease throughout time of TFVAs was observed (Fig. 1A). A non-parametric Spearman test was used to verify this observation (Table 1). Since VFAs longer than butyric acid were mostly not present or only in very low concentrations (Additional file 1), this analysis was limited to acetic, propionic, butyric acid and the sum of all VFAs (TVFA). No significant trend was observed for BGP4, BGP6 and BGP7. BGP3 and BGP5 indicated a decrease in VFAs, but did not provide enough data points for a reliable comparison. A significant decrease was observed for BGP1 and BGP2. In order to increase comparability and also to include BGP3 and BG5 in the significance analysis, all seven systems were treated as a data cloud in a joint analysis. For this, VFA concentrations of all 7 biogas plants were initially normalized to a value between 0 and 1. The values of all 7 systems were then averaged and treated as a data cloud. The resulting data cloud was then subjected to a non-parametric Spearman analysis. The trend was clear and significant for acetic acid and propionic acid (Fig. 2A and B). For butyric acid, only a slight—but yet significant—decrease was observed (Fig. 2C).

**Table 1:** Correlation of VFAs over time applying a nonparametrical Spearman test

	TVFAs		Acetic acid		Propionic acid		Butyric acid	
	*R*^2^	*p*-value	*R*^2^	*p*-value	*R*^2^	*p*-value	*R*^2^	*p*-value
BGP1	0.4807	< 0.0001****	0.5663	< 0.0001****	0.2558	< 0.0001****	1.000	na
BGP2	0.4849	< 0.0001****	0.4633	0.0003***	0.5118	< 0.0001****	0.3455	0.0001****
BGP3	0.7677	na	0.7617	na	0.7349	na	0.7349	na
BGP4	0.3696	0.1041ns	0.1058	0.1967ns	0.1627	0.6441ns	1.000	na
BGP5	0.9505	na	0.9504	na	0.9456	na	1.000	na
BGP6	0.0350	0.1965ns	0.0100	0.4650ns	0.1104	0.0924ns	0.0272	0.3847ns
BGP7	0.0011	0.7833ns	0.0091	0.9500ns	0.0007	> 0.9999ns	1.000	na

Several results were not significant (ns). The significance level was not determined for fewer than three values (na)

The observed decrease in acetic, propionic, and butyric acid concentrations is in accordance with existing literature. A recent study at laboratory scale demonstrated that conductive materials help to lower the concentrations of propionic and butyric acids, and explained that this phenomenon is due to an increased rate of DIET, which in turn reduced the amount of inhibiting hydrogen [7]. In this context, the falling concentrations of organic acids could be interpreted as an indication for DIET, even if the observation of the acids alone is not sufficient for this. It must be taken into account that other mechanisms besides DIET must now also be considered. In a recent study, it was shown that carbon nanotubes had a positive effect on methane formation without the DIET being detected [18].

The organic loading rate for all digesters is shown in Table 2, and all plant operators confirmed that the respective loading rate was maintained throughout the study. Therefore, the reduced concentration of organic acids cannot be explained by a change of loading rate.

**Table 2:** Overview on digester systems: all systems were mesophilic continuous stirred tank reactors (CSTRs)

Anaerobic digester	Substrates	Loading rate [kg VS m^−3^ d^−1^]	Retention time in days [d]	Volume of all fermenters excluding the digestate storage [m^3^]
BGP1	Corn silage, poultry manure, cereal silage, sugar beet, swine manure, grain kernel (mashed)	2.9	117	7.700
BGP2	Corn silage, poultry manure, grass silage, sugar beet	3.1	113	7.000
BGP3	Corn silage, grain kernel (mashed), perennial rye, cereal silage, cattle manure, cattle slurry	2.7	85	2.900
BGP4	Corn silage, cereal silage, cup plant, swine slurry, cattle manure	4.3	54	3.200
BGP5	Cattle manure, corn silage, poultry manure, swine slurry	2.1	105	4.600
BGP6	Corn silage, cereal grain (mashed), poultry manure, cattle manure with feed residues	3.2	95	2.600
BGP7	Corn silage, cereal silage, grass silage, sugar beet, cereal grains (mashed)	2.3	153	4000

### F420-fluorescent microscopy upon biochar addition

**Fig. 3:**
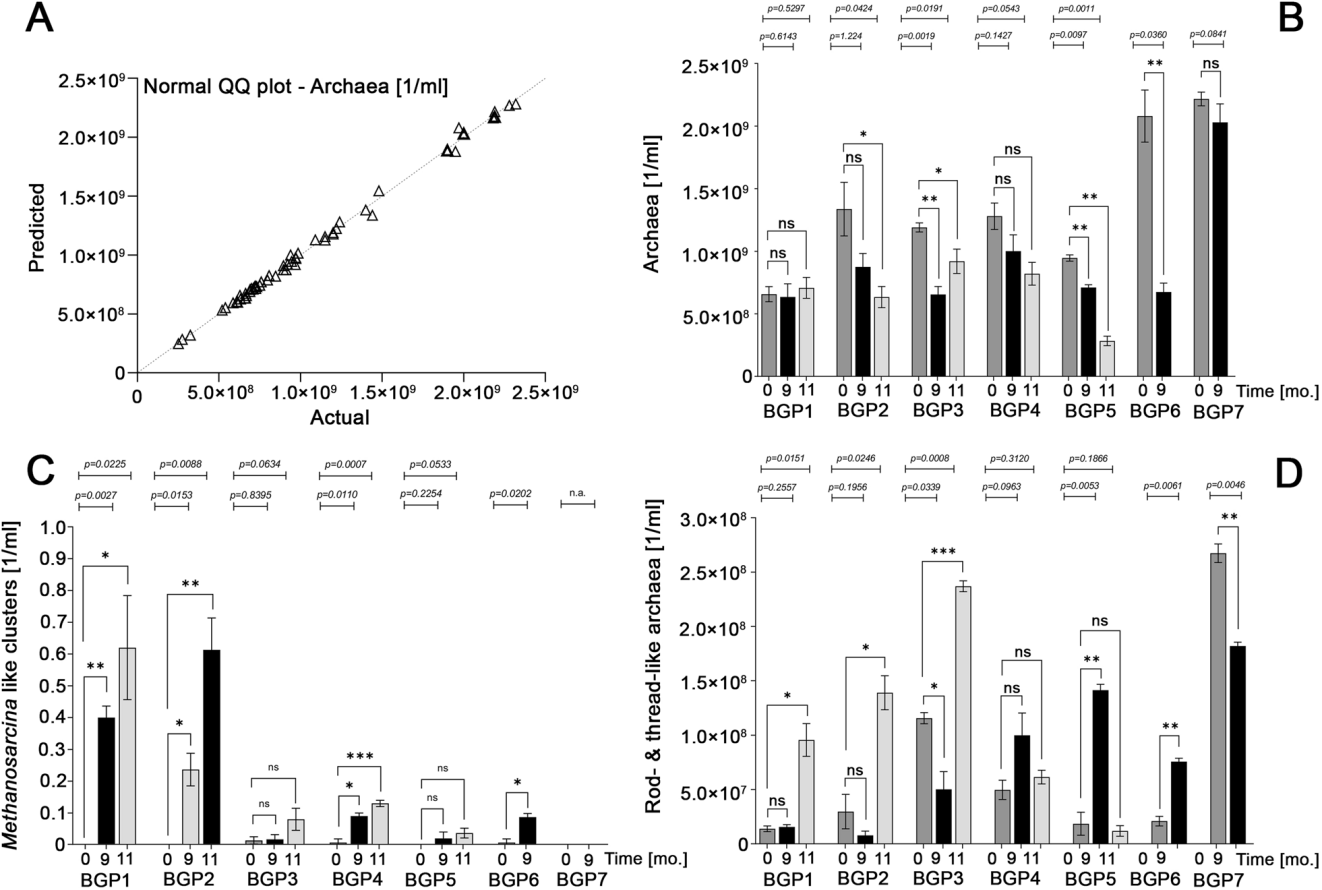
Quantification of methanogenic archaea before and after supplementation: The cofactor F420 was used to count methanogenic archaea using epifluorescent microscopy. The QQ plot resulting from the Shapiro–Wilk test (**A**) is shown as an example for the counting of all archaea (**B**), but it was also carried out for the counting of *Methanosarcina*-like clusters (**C**) and rod-shaped and thread-like archaea (**D**). Analysis was performed before biochar was added, and 9 and 11 months after supplementation. Each bar shows a mean value of 48 pictures taken from three different slides. A two-tailed paired t-test was applied to assess significance

Before biochar was applied to BGP1-BGP7, all plant operators provided fresh sludge samples for the analysis of methanogenic archaea based on the cofactor F420. After 9 and 11 months, all plant operators provided further samples for F420 analysis. F420 signals were counted using the ImageJ software (Fig. 3). In general, the detected concentration of methanogenic archaea was in a similar range as in other studies [19, 20]. Interestingly, the number of methanogenic archaea seemed to decrease slightly throughout time upon the addition of biochar. Although the decrease was not observed for all timepoints (BPA1 behaved different) and samples for BGP6 and BGP7 were not accessible during month 11, a two-tailed paired t-test revealed a significant decrease of the archaea number for BGP2, BGP3, BGP5 and BGP6. Although not significant, BGP4 and BGP7 showed a decrease in methanogenic archaea as well (Fig. 3B).

Regarding methanogenic phenotypes, mainly cocci were observed. In a recent study, conductive particles led to an increase in the ratio of acetoclastic methanogens [10]. Acetoclastic methanogens that are typically involved in anaerobic digestion processes are *Methanothrix* and *Methanosarcina* [21, 22]. However, typical phenotypes for *Methanothrix* (thread-like) or *Methanosarcina* (sarcina-like cluster) were scarcely detected. On average, less than one *Methanosarcina* cluster was detected per picture (Fig. 3C). Although this number is very small, it is interesting that all of the plants tested, with the exception of BGP7, showed an increase in the number of *Methanosarcina*-like clusters and some of them were significant. No clear trend was observed for rod-like and thread-like phenotypes (Fig. 3D). As the hydrogenotrophic methanogen *Methanoculleus* (coccus shape) is usually enriched in continuous stirred tank reactors [21], and mainly methanogenic cocci were detected in the analyzed digesters, our results suggest that *Methanoculleus* was also prevalent in the present study. In the case of BGP1, this assumption was verified by 16S-rRNA gene amplicon high-throughput sequencing (Fig. 6). Under the assumption that supplemented biochar increased the rate of DIET, our results suggest that hydrogenotrophic methanogens could be involved in DIET. In concordance with this hypothesis, recent studies have suggested that DIET is more widespread than previously thought, and that DIET is not only restricted to acetoclastic methanogens. To give some examples: recently, the first methanogen able to produce electrically conductive pili was detected, and identified as the hydrogenotrophic *Methanospirillum hungatei* [13]. Another recent study suggested that the hydrogenotrophic *Methanobacterium* is able to perform DIET [14]. Regarding *Methanoculleus*, several species have been tested in vitro, but were not able to grow in syntrophic co-culture with the typical electrogenic bacterium *Geobacter metallireducens* [23].

However, the observed results could also be explained using other mechanisms. In a study published in 2017, a positive effect was found using carbon nanotubes without indications of DIET. The realization that mechanisms other than DIET could be involved is particularly interesting because no DIET has yet been detected for the hydrogenotrophic methane generator *Methanoculleus* (as already described above). The observation that carbon nanotubes exerted a stronger effect on hydrogenotrophic methanogens than on acetoclastic methanogens and this presumably without DIET therefore opens up a different perspective for the results presented here and is in accordance with the high proportion of *Methanoculleus* among the methanogens. The exact mechanism triggered by carbon nanotubes is not yet fully understood, but it has been described that they increasingly shift the redox potential into the negative.

### Fluorescence microscopy with ground biochar particles

**Fig. 4:**
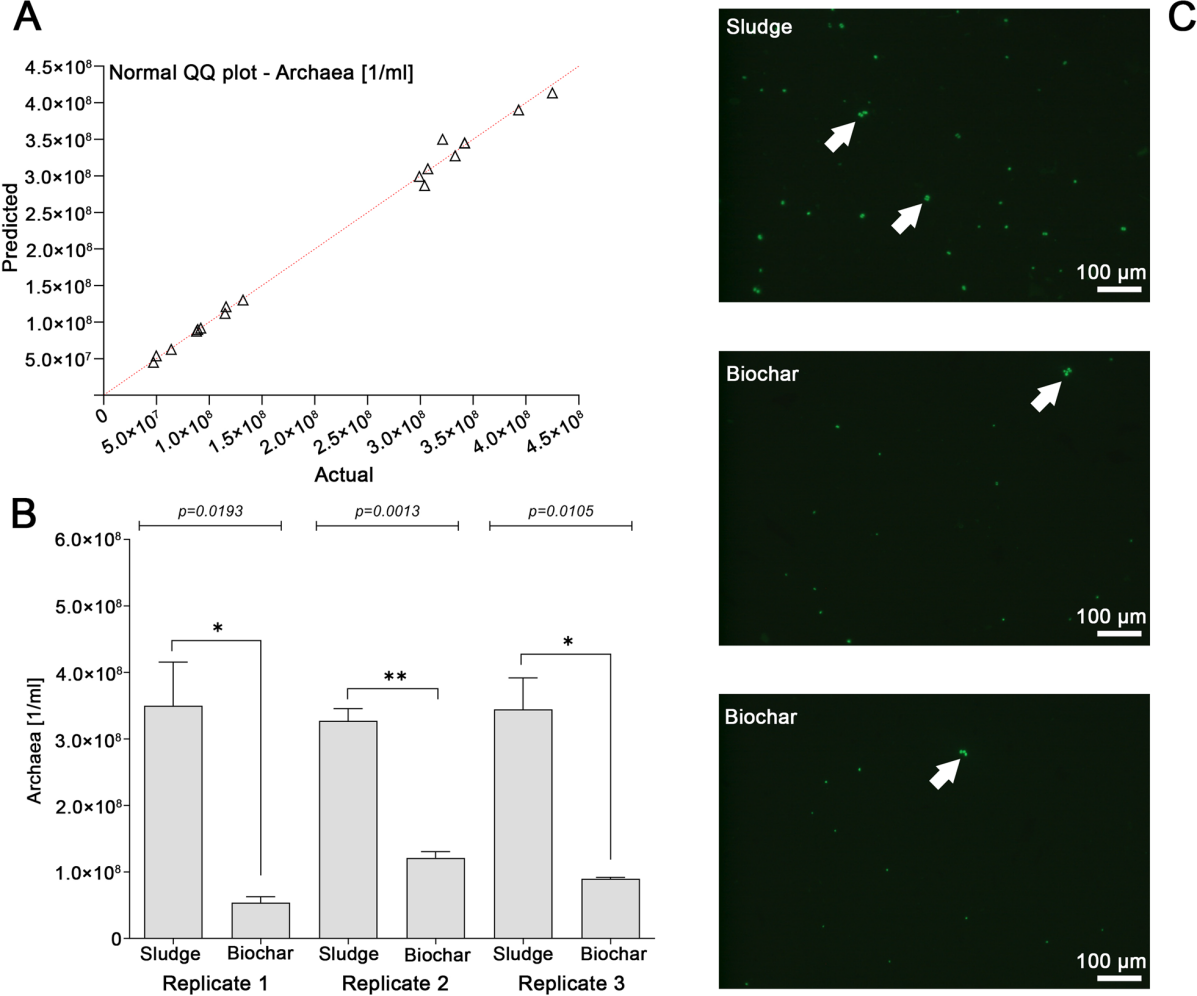
Methanogenic archaea found in biochar particles: after applying the Shapiro–Wilk test (**A**), a two-tailed paired t-test was used to assess significance for all archaea. Number of methanogenic archaea based on the quantification of cofactor F420 signals (**B**). Biochar particles from BGP1 were collected from the digestate immediately after it left the digester. Pictures of methanogenic archaea found in biochar particles (**C**). *Methanosarcina*-like clusters are highlighted with white arrows

The fluorescence microscopy results shown in Fig. 3 were performed with diluted sludge as described in Material and methods. The counting of methanogenic archaea in the defined volume of sludge does not take into account those archaeal cells, which are immobilized in the biochar. Therefore, further experiments were performed focusing on a defined weight of ground biochar particles. The plant operator of BGP1 provided access to several tons digestate, which left the reactor exactly before the sampling. Two falcon tubes were filled with biochar particles, which were collected directly from the digestate. In a first analysis, biochar particles were ground to powder and resuspended in 1 ml of PBS buffer per 1 g of powder. Upon inverting, the samples were analyzed using fluorescence microscopy (Fig. 4).

Although the number of methanogenic archaea was much lower in the ground biochar powder compared to the fresh sludge (Fig. 4A), ground biochar particles clearly contained methanogenic archaea (Fig. 4B). Biochar samples which were not inserted into the digesters did not show F420-signals. As previously described for highly viscous sludge from continuous stirred tank reactors [21], very little *Methanosarcina*-like clusters were found, which was also the case in BGP1–BGP7 (Fig. 3C). Still, a few *Methanosarcina* were observed, even in the ground biochar, suggesting that the biochar pores were big enough for such cluster-forming methanogens (Fig. 4C). The majority of the observed methanogens were cocci, suggesting that the same methanogens were present in both the biochar and the sludge.

### Analysis of taxonomic profiles of ground biochar particles at phylum level

**Fig. 5:**
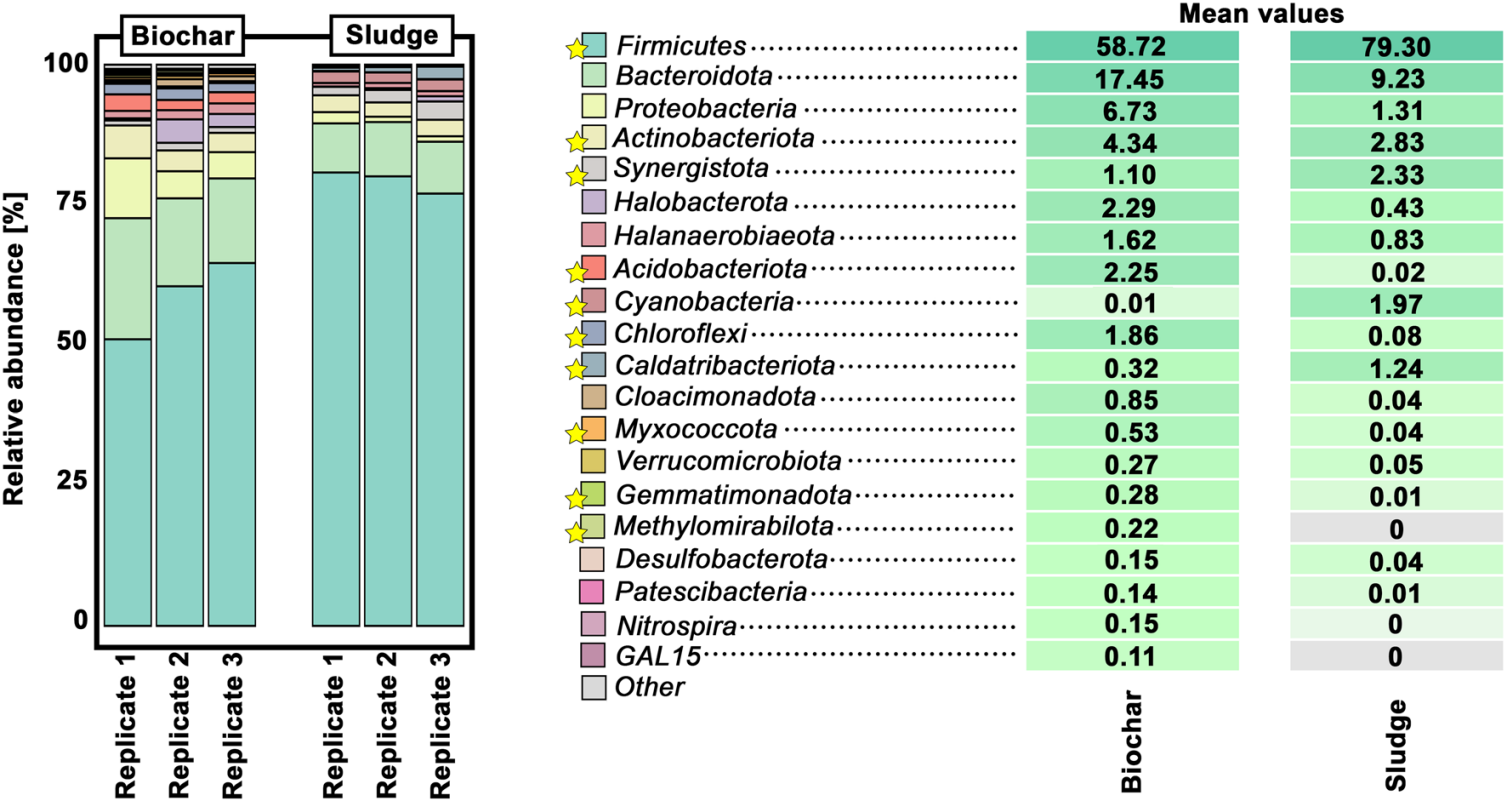
Taxonomic profiles in sludge and biochar from BGP1 at phylum level, obtained through 16S-rRNA gene amplicon high-throughput sequencing. For the sake of simplicity, only the most abundant phyla are shown. Differences in the mean values that are significant are highlighted by yellow stars in the legend. Significances were analyzed using the DESeq2 differential abundance analysis [46] and *p*-values were < 0.05

To obtain a more detailed insight into the taxonomic profiles present in the sludge and in the biochar particles from BGP1, 16S-rRNA gene amplicon high-throughput sequencing was performed. The main phyla present in all samples were *Firmicutes* (~ 69%), *Bacteroidota* (~ 13%) and Proteobacteria (~ 4%) (Fig. 5).

The ground biochar samples displayed a higher—yet not significant—relative abundance of *Bacteroidota* and a lower relative abundance of *Firmicutes* (FDR adjusted *p*-value < 0.05; DESeq2 test) in comparison to the digester sludge samples. *Firmicutes* are well-known degraders of plants and complex carbohydrates [24]. The lower ratio of Firmicutes in the biochar samples might indicate that bacteria within biochar particles are rather associated with secondary fermentation (acetogenesis) than with hydrolytic and acidogenic events. Our results also revealed that biochar powder contained higher relative abundances of *Acidobacteria*, *Halanaerobiaeota*, *Halobacterota* and *Proteobacteria*, although only *Acidobacteria* changed significantly. This suggests that biochar particles are subjected to more stressful conditions: *Acidobacteria* are described as robust and adapted to stressful conditions in soil [25]; *Halanaerobiaeota* and *Halobacterota* are generally known to be associated with high salt contents and their higher abundance might be explained by the adsorptive characteristics of biochar; and *Proteobacteria* are associated with nitrogen and ammonium metabolism [26] and, therefore, their increased abundance in the biochar might be explained due to precipitation of ammonia within the biochar. Altogether, our results indicate that adsorptive characteristics of biochar particles can lead to locally increased concentrations of salt and other inhibitors, which in turn has a strong impact on the underlying taxonomic profile. At this point, it should be considered that adsorption effects, DIET and other effects are difficult to distinguish. A study from 2017 investigated the possibility of adsorption-based extraction of organic acids and indicated that the adsorption of organic acids by numerous interfering ions such as Na^+^, K^+^, H_2_PO_4_^−^/HPO_4_^2−^, Cl^−^, and SO_4_^2−^ is problematic [27]. There was no activation process of the carbon used in the present work, so that the adsorption capacity is lower than for activated carbon. If the observed effects were mainly caused by adsorption, one could have expected a significant reduction in the concentrations of organic acids in all anaerobic digester plants. However, there was no decrease in organic acids in BGP4, BGP6 and BGP7. Nevertheless, it cannot be ruled out that adsorption-based processes had an influence on observed effects, which could also be responsible for the higher relative abundance in biochar of microorganisms involved in secondary fermentation processes. In this relation, it must be noted that the phylum *Chloroflexi* showed a significant higher abundance in the biochar powder (FDR adjusted *p*-value < 0.05; DESeq2 test). In a previous report, an enrichment of *Chloroflexi* in anaerobic biofilms was described [28]. It has also been reported that *Chloroflexi* can be involved in syntrophic relations [29, 30]. In relation to the aforementioned decrease of* Firmicutes*, this supports the hypothesis that biochar particles are particularly involved in syntrophic degradation processes. On the other hand, *Cyanobacteria* were overrepresented in the sludge samples (FDR adjusted *p*-value < 0.05; DESeq2 test). It has to be noted that the sequencing reads assigned to *Cyanobacteria* could correspond partially to chloroplasts, which are an indicator of undegraded plant biomass. Less than 1% of the reads represent chloroplasts (PCC-6307). The remaining reads (2%), which were assigned as *Cyanobacteria*, are represented by the genus *Cyanobium (data not shown)*. This phenomenon has been previously reported for a lab-scale reactor, which was fed with fresh grass biomass and a high ratio of* Cyanobacteria* was observed [28].

### Analysis of taxonomic profiles of ground biochar particles at genus level

The most abundant genera detected in both sets of samples were *Limnochordia MBA03* (36% in biochar samples and 46% in sludge), *Proteiniphilum* (14% and 7%), *Caldicoprobacter* (5% and 8%) and *Amphibacillus* (2% and 4%).

The frequency *of Limnochordia MBA03* is of special interest, since this genus was observed in a cathodic enrichment culture in 2018 [31]. In a recent article, in which 20 biogas plants were compared, this organism was observed together with *Methanosarcina*, and a syntrophic relationship has been suggested between both of them [32]. The fact that *Limnochordia MBA03* occurs both in the biochar particles and in the liquid phase could indicate that the biochar particles can also be used as conductive structures by microorganisms in the liquid phase. At this point, however, it cannot be ruled out that *Limnochordia MBA03* also grows on other conductive structures or even without conductive structures, as this genus was also abundant in anaerobic digesters not treated with conductive particles [32]. Besides *Limnochordia MBA03, the* genus *Proteiniphilum* is another hint for electroactivity as this genus has been described within electroactive consortia [33]. *Proteiniphilum* is known to grow on nitrogen rich substrates (e.g., yeast, peptone). In the case of *Proteiniphilum acetatigenes*, this species is unable to grow on multiple carbohydrates, alcohols and fatty acids [34], suggesting an intense nitrogen metabolism within biochar particles. This might be explained by the fact that poultry manure, known for its high nitrogen content, was among the substrates that were fed into BGP1 (Table 2).

Regarding the sludge samples, the ratio of *Proteiniphilum* was much lower in comparison to the biochar samples. On the other hand, the sludge samples displayed much higher ratios for *Caldicoprobacter*, a genus known to grow with high ammonium concentrations [35]. A reason for the shift from *Caldicoprobacter* to *Proteiniphilum* might be a local enrichment of ammonia in the biochar particles, which *Proteiniphilum* might tolerate better than *Caldicoprobacter.* Another explanation could be that nitrogen metabolism by* Proteiniphilum* was supported by electroactivity by electroactivity in the biochar particles, as it is well known that several amino acids are degraded in syntrophic relations [8]. It has been previously postulated that *Caldicoprobacter* is involved in syntrophic oxidation processes, but this has not yet been brought into connection with electroactivity [36]. Therefore, it could be possible that biochar particles increased the rate of DIET during nitrogen metabolism, which in turn caused a shift from *Caldicoprobacter* to *Proteiniphilum.*

**Fig. 6:**
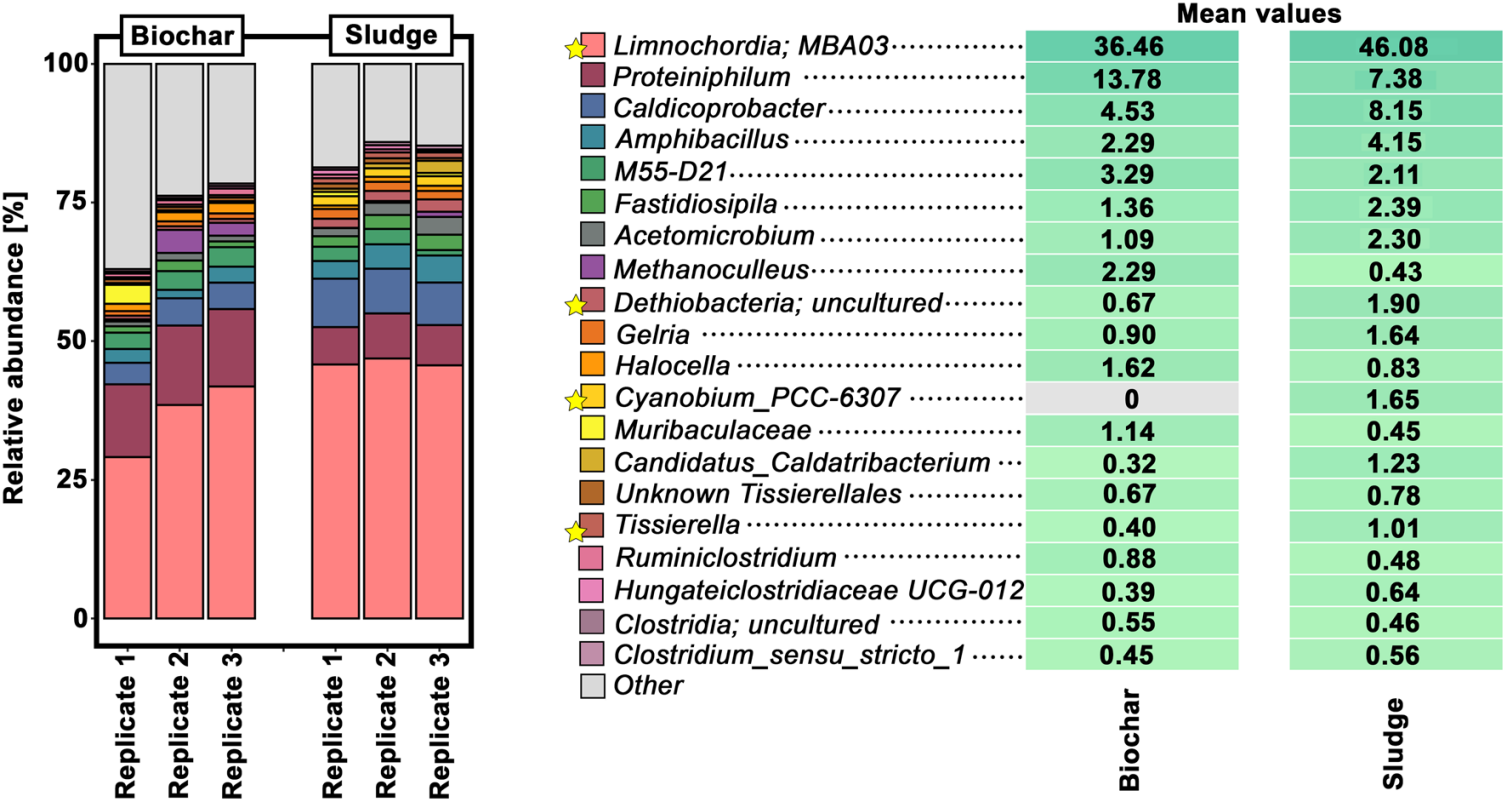
Taxonomic profiles in sludge and biochar from BGP1 at genus level and obtained through 16S-rRNA gene amplicon high-throughput sequencing. For the sake of simplicity, only the most abundant genera are shown. Differences in the mean values that are significant are highlighted by yellow stars in the legend. Significances were analyzed using the DESeq2 differential abundance analysis [46] and *p*-values were < 0.05

Although bacteria-specific primers were used (as described in Material and methods), several archaea were recorded. One methanogen (*Methanoculleus*) was even among the most abundant prokaryotic genera (Fig. 6). The fact that mainly *Methanoculleus* was found is in accordance with above-described microscopic results, where mainly cocci were found. Taking into account that applied biochar particles may increase the rate of DIET, our results suggest that *Methanoculleus* may be involved in DIET. However, since *Methanoculleus* has not been described as capable to perform DIET so far, this needs to be further studied. Although the total number of methanogenic archaea found in biochar samples was lower than the number of archaea found in sludge—as measured by fluorescence microscopy—the relative abundance of *Methanoculleus* was higher in the biochar particles (2%) than in the sludge (less than 1%), supporting the previous hypothesis that biofilms on biochar particles are more involved in secondary fermentations steps (in syntrophic relation with methanogenesis).

### Microbial diversity on biochar particles is increased

**Fig. 7:**
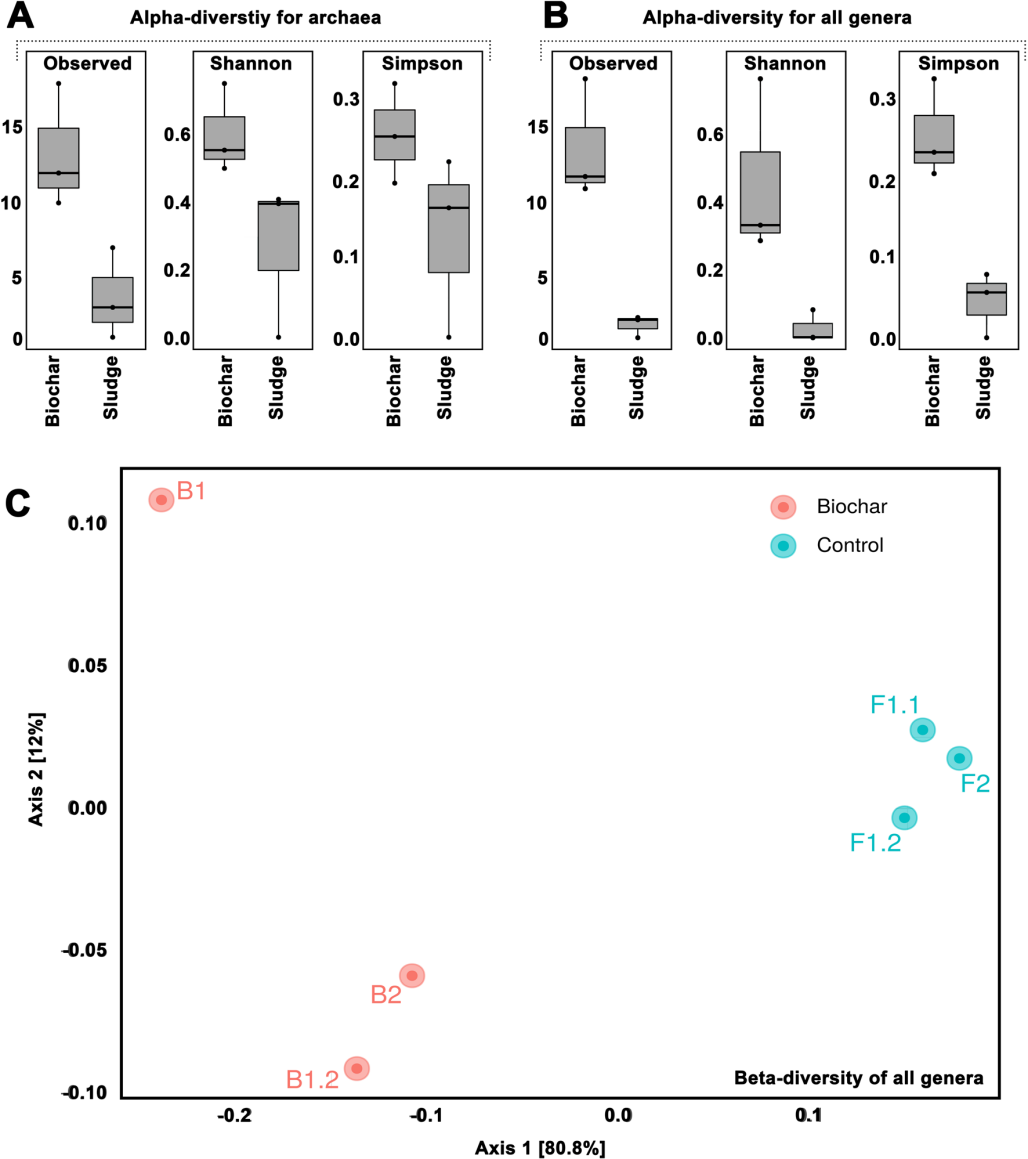
Microbial diversity in biochar particles on genus level: **A** α-diversity of archaea according to richness (Observed) and diversity indices (Shannon and Simpson); **B** α-diversity of all genera according to richness (Observed) and diversity indices (Shannon and Simpson); C β-diversity of all genera represented through a principal coordinates analysis

To investigate whether microbial diversity differed between biochar particles and general sludge, the α- and β-diversity of both groups of samples were calculated (Fig. 7). The β-diversity is shown in a principal component analysis (PCoA) and indicates that the microbial communities of sludge samples and powdered biochar are substantially different from each other. Regarding archaea, the biochar samples analyzed in this work did not only display a higher relative abundance of *Methanoculleus* (Fig. 6), but also a higher α-diversity of methanogenic archaea (Fig. 7A). Interestingly, this increased diversity was also observed when considering all prokaryotic genera (Fig. 7B). There are several reasons, which might explain these observations. For example, the porous surface could facilitate biofilm formation, and adsorption might influence the microbial community as well. Due to adsorption, a local enrichment of salt and inhibitors might cause very harsh conditions in the biochar particles, forcing the involved microorganisms to continuously adapt. Although one might expect to obtain a lower diversity under harsh or even extreme conditions, some authors describe high diversities under extreme conditions. For example, it has been described that numerous alkaline and hypersaline environments show high microbial diversity, and that the adaptive mechanisms under extreme conditions can enable very useful capabilities, such as a “*control of membrane permeability, control of intracellular osmotic balance, and stability of the cell wall, intracellular proteins, and other cellular constituents*” [37].

Based on the aforementioned observations, it is possible to hypothesize that digester sludge provides a diverse microbial community, that is forced to develop adaptive mechanisms once they come into contact with the respective biochar particles.

The aforementioned assumption that salt and inhibiting compounds are enriched in biochar particles is in agreement with the existing literature. For example, a recent study described that 5 different biochar types, which were evaluated as supplements for anaerobic digestion, retained Fe, Co, Ni and Mn [38]. Also, the potential enrichment of functional microbes has been previously suggested, particularly with respect to the stimulation of the secretion of extracellular polymeric substances (rapid sludge granulation), increased microbial abundance and improvement of DIET [39].

Interestingly, other authors have described an enrichment of *Sporanaerobacter* and *Enterococcus*, *Methanosarcina* [40] and *Methanothrix* [41] on biochar. In the present study, none of these genera were enriched. A reason for this difference might be that the biochar surface and the inner region of the biochar particles can be colonized differently. Although many of the articles discussed in a recent review [39] highlight an enrichment of *Methanosarcinales*, it is also mentioned that these species grow especially on the surface of biochar particles. In contrast, the inner regions might promote the growth cocci such as *Methanoculleus*, which are smaller than the threadlike or cluster-forming *Methanosarcinales* [39].

## Conclusions

The use of biochar in industrial biogas plants caused significant changes in the concentration of organic acids as well as in taxonomic profiles. Even if the reduction in the concentration of organic acids could be interpreted as an indication of DIET, this interpretation contradicts the high abundance of the genus *Methanoculleus*, which has not yet been associated with DIET. As indicated in another study [16], another, previously unknown mechanism could be involved.

While caution should be exercised based on microscopic counts, epifluorescent microscopy indicated a shift in the number of methanogenic archaea, suggesting that there is a decrease in methanogenic cell numbers in sludge and an increase in the respective biochar particles. One of the digesters was analyzed in more detail by 16S-rRNA gene amplicon high-throughput sequencing, comparing the taxonomic profiles in the sludge and in hand-picked biochar particles from fresh digestate. The taxonomic profile in the biochar particles substantially differed from the one observed in the sludge samples, and this profile suggested an increased electroactivity associated to the biochar particles, as well as an increased biodiversity, which should be characterized in depth in future studies.

## Materials and methods

### Analyzed digesters and biochar supplementation

F420 fluorescence microscopy was used to investigate the effect of biochar on the absolute cell count of methanogenic archaea in biogas plants. In order to increase the trustworthiness and to guarantee reproducibility, seven systems were tested in parallel. Seven German anaerobic digesters plants were supplemented with biochar over a duration of one year. An overview of the respective digester plants is given in Table 2. All digester systems analyzed were industrial continuous stirred tank reactors and it must be noted that several of them were in a problematic state, indicated by high concentrations of acetic acid. All digester systems were supplemented with 1.8 kg of biochar per t of reactor content (“*Carboferm*”, a wood-based capillary charcoal from the LUCRAT GmbH). All fermenter systems were supplemented with 1.8 kg of biochar per t of reactor content. The biochar was gradually added to the fermenter over 12 days. Then the substrate of the respective plants was premixed in a ratio of 1.8 kg of biochar per t of substrate, with the feeding and loading rate of the respective plants being maintained (Table 2).

### Analysis of organic acids

Biogas productivity could not be measured during the experiments. Sporadically, chemical parameters were recorded by the companies T&B—Die Biogasoptimierer GmbH (Tarp. Germany) and WESSLING GmbH (Altenberge, Germany). These companies recorded total solids (TS), volatile solids (VS), content of ammonia (NH_4_-N), pH, spectrum of organic acids and the volatile organic acid and buffer capacity ratio (FOS/TAC). Only the content of TVASs, acetic acid, propionic and butyric acid provided useful information for the present study (Figs. 1 and 2, Table 1), and the respective raw data are recorded for each plant (Additional file 1).

### Quantifying the cofactor F420

Involved digester plants sent their samples by overnight mail order to the Robert Boyle Institute (Jena, Germany), and samples were analyzed upon receipt. For this, samples were diluted 1:10 with an anti-fading solution (RotiR-Mount FluorCare, Carl-Roth, Germany), and 3 μL of the diluted sample were pipetted between the cover slip and the slide. An epifluorescent microscope (Axio Lab.A1, Carl Zeiss, Germany) was used to quantify cofactor F420 as an indirect measure of methanogenic archaea load. The methodology was similar to a recent study from Hardegen et al. [19]. Excitation occurred with wavelengths ranging from 400 to 440 nm. Emitted light with wavelengths between 500 and 550 nm was collected and analyzed using the ImageJ-Software (400 Å ~ magnification and 126 ms exposure time). For each sample, 48 pictures were analyzed. In total, samples from three time points were collected: (1) control before adding biochar, (2) after 9 months of supplementation with biochar, and (3) after 11 months of supplementation with biochar.

From all seven digesters plants, one plant provided access for further analysis (BGP1). To get a deeper insight into the taxonomic profile of the anaerobic digester microbiome, samples were taken from the digester liquor, and biochar fragments from fresh digestate were collected manually. Upon the sampling, the digester liquor was analyzed under the microscope as described above. Biochar fragments were ground and resuspended in PBS buffer (1 g of powdered biochar per 1 ml of PBS). In order to make the results per sludge volume and weight of the biochar powder comparable, the volume of the biochar was also determined. For this purpose, the liquid displaced by the biochar was determined by reverse pipetting. The density of the biochar amounted to 0.51 g ml^−1^ (± 0,08 g ml^−1^). After vortexing, 2 µl of the resuspended biochar powder was pipetted between the cover slip and the slide. Following this, the cofactor F420 was analyzed as previously described. Additionally to the fluorescent microscopy, liquid samples and biochar fragments were fixed in 50% ethanol for subsequent DNA analysis (16S-rRNA amplicon gene high-throughput sequencing).

### 16S-rRNA gen amplicon high-throughput sequencing

Primers 341F (5′ CCT AYG GGR BGC ASC AG 3′) and 806R (5′ GGA CTA CNN GGG TAT CTA AT 3′) were used to amplify the V3–V4 region of the 16S rRNA gene for prokaryotes. All PCR reactions were carried out with Phusion® High-Fidelity PCR Master Mix (New England Biolabs). PCR products were mixed at equal density ratios. The pool was then purified with Qiagen Gel Extraction Kit (Qiagen, Germany). Sequencing libraries were generated with NEBNext® UltraTM DNA Library Prep Kit for Illumina and quantified via Qubit and q-PCR. Finally, the NovaSeq 6000 Sequencing System (2 × 250 bp) was employed for sequencing the samples. All sequence data are stored in the Sequence Read Archive (SRA) of the National Center for Biotechnology Information (NCBI; Bioproject: PRJNA727077).

### Bioinformatic analysis

Raw Illumina sequences were analyzed using Qiime2 (v. 2020.8) [42]. Briefly, the quality of the reads was assessed with the Demux plugin, and the sequences were subsequently corrected, trimmed and clustered into amplicon sequence variants (ASVs) via DADA2 [43]. The taxonomy of each sequence variant was assigned employing the classify-Sklearn module from the feature-classifier plugin. SILVA (v. 138) was used as reference database for 16S rRNA alignment [44]. It is worth highlighting that SILVA's nomenclature was used for taxonomy (i.e. Bacteroidota was used instead of Bacteroides). Phyloseq package was employed for analyzing the data [45]. All the α-diversity tests were carried out using ASVs and rarefying to the lowest library size (= 115,626 seqs). DESeq2 was used for differential abundance analyses [46].

## Supplementary Information

**Additional file 1.** Recorded raw data for acetic-, propionic-, and butyric acid.

## Data Availability

All sequence data are stored in the Sequence Read Archive (SRA) of the National Center for Biotechnology Information (NCBI; Bioproject: PRJNA727077).
